# Impact of COVID-19 on notifiable diseases: a time series study

**DOI:** 10.1590/1980-220X-REEUSP-2024-0098en

**Published:** 2025-02-17

**Authors:** Pollyanna Kassia de Oliveira Borges, Camila Marinelli Martins, Caroliny Stocco, Jean Fernando Sandeski Zuber, Wesley Sousa Borges, Erildo Vicente Muller, Milene Zanoni da Silva, Carlos Eduardo Coradassi, Mariele Katherine Jungles, Eliseu Alves Waldman

**Affiliations:** 1 Universidade de São Paulo, Faculdade de Saúde Pública, Departamento de Epidemiologia, São Paulo, SP, Brazil.; 2 Universidade Estadual de Ponta Grossa, Departamento de Saúde Pública, Ponta Grossa, PR, Brazil.; 3 Fundação Municipal de Saúde de Ponta Grossa, Vigilância Epidemiológica, Ponta Grossa, PR, Brazil.; 4 Centro de Testagem e Aconselhamento, Ponta Grossa, PR, Brazil.; 5 Centro Universitário Unicesumar, Departamento de Ciências da Saúde, Ponta Grossa, PR, Brazil.

**Keywords:** Public Health Surveillance, COVID-19, Dengue, Tuberculosis, Pulmonary, Syphilis, Vigilancia en Salud Pública, COVID-19, Dengue, Tuberculosis Pulmonar, Sífilis, Vigilância em Saúde Pública, COVID-19, Dengue, Tuberculose Pulmonar, Sífilis

## Abstract

**Objective:**

To assess the indirect impact of the COVID-19 pandemic on tuberculosis, congenital syphilis, gestational syphilis, and dengue.

**Method:**

Epidemiological, ecological, time series study. The period from 2015–2021 was analyzed, in a medium-sized municipality in Paraná, and compared to the state of Paraná and Brazil. Data were extracted from the Notifiable Diseases Information System (*SINAN*). Gross and standardized incidence/detection rates were calculated. Temporal trends were constructed using linear regression models.

**Results:**

There was an increase in the rates of congenital syphilis in 2021 (30% of cases, β = 4.47, 95% CI: 1.24–7.69), gestational syphilis during the pandemic (41% of cases, β = 3.65, 95% CI: 1.08–6.21), and tuberculosis (β = 2.48, 95% CI: 1.08–3.88). There was an increase in the standardized mean rate for tuberculosis (p = 0.022) and congenital syphilis (p = 0.034) in the first two pandemic years.

**Conclusion:**

COVID-19 indirectly impacted the control of tuberculosis, congenital and gestational syphilis in the municipality studied. The high rates did not follow the national and state trend and indicate that health surveillance should be municipalized for local priorities.

## RESUMO

**Objetivo:**

Avaliar o impacto indireto da pandemia da COVID-19 sobre a tuberculose, sífilis congênita, sífilis gestacional e dengue.

**Método:**

Estudo epidemiológico, do tipo ecológico, de série temporal. Foi analisado período de 2015–2021, em município de médio porte do Paraná, e comparado ao estado do Paraná e Brasil. Dados foram extraídos do Sistema de Informações de Agravos de Notificação. Calcularam-se taxas brutas e padronizadas de incidência/detecção. Tendências temporais foram elaboradas com modelos de regressão linear.

**Resultados:**

Verificou-se aumento nas taxas de sífilis congênita em 2021 (30% dos casos, β = 4,47, IC 95%: 1,24–7,69), sífilis gestacional durante a pandemia (41% dos casos, β = 3,65, IC 95%: 1,08–6,21) e tuberculose (β = 2,48, IC 95%: 1,08–3,88). Houve crescimento da taxa média padronizada para tuberculose (p = 0,022) e sífilis congênita (p = 0,034) nos dois primeiros anos pandêmicos.

**Conclusão:**

A COVID-19 impactou indiretamente no controle da tuberculose, sífilis congênita e gestacional no município estudado. As taxas elevadas não seguiram a tendência nacional e estadual e indicam que a vigilância em saúde deve ser municipalizada para as prioridades locais.

## RESUMEN

**Objetivo:**

Evaluar el impacto indirecto de la pandemia de COVID-19 sobre la tuberculosis, la sífilis congénita, la sífilis gestacional y el dengue.

**Método:**

Estudio epidemiológico, ecológico, de series de tiempo. Se analizó el período de 2015–2021, en un municipio de tamaño mediano de Paraná, y se comparó con el estado de Paraná y Brasil. Los datos se extrajeron del Sistema de Información de Enfermedades de Notificación Obligatoria. Se calcularon las tasas de incidencia/detección brutas y estandarizadas. Las tendencias temporales se crearon con modelos de regresión lineal.

**Resultados:**

Hubo un aumento en las tasas de sífilis congénita en 2021 (30% de los casos, β = 4,47, IC 95%: 1,24–7,69), sífilis gestacional durante la pandemia (41% de los casos, β = 3,65, 95% IC: 1,08–6,21) y tuberculosis (β = 2,48, IC 95%: 1,08–3,88). Hubo un aumento en la tasa promedio estandarizada de tuberculosis (p = 0,022) y sífilis congénita (p = 0,034) en los dos primeros años de la pandemia.

**Conclusión:**

La COVID-19 impactó indirectamente en el control de la tuberculosis, sífilis congénita y gestacional en el municipio estudiado. Las altas tasas no siguieron la tendencia nacional y estatal e indican que la vigilancia de la salud debe ser municipalizada para las prioridades locales.

## INTRODUCTION

The COVID-19 pandemic has directly and indirectly impacted health events and services. Positive or negative changes in morbidity and mortality, not associated with SARS-CoV-2 infection and its complications, are considered indirect impacts of COVID-19. Indirect effects may or may not coincide with the waves of the pandemic^(1)^. The indirect repercussions on priority agendas of the Brazilian Public Health System (*SUS*) are being studied and scientifically disseminated after the emergency situation^(2)^.

Surveillance of notifiable diseases was indirectly impacted by the pandemic^(3)^. There was a redirection of human resources to face COVID-19. Public health laboratories prioritized COVID-19 testing and may have delayed diagnoses of other notifiable diseases. Data collection, epidemiological investigations, and closure of non-COVID-19 cases may have been reduced. Social distancing and fear of SARS-CoV-2 infection have led to a decrease in the sensitivity of health surveillance systems for notifiable diseases. Prevention and immunization to control these diseases were suspended or reduced due to the surveillance system overload or lower adherence by the population. Health communication with the population prioritized COVID-19 over other notifiable diseases, whose continuous monitoring and incidence rates were indirectly affected by combined factors.

Knowing the indirect impacts of the pandemic on notifiable diseases helps to understand the patterns of occurrence and mortality of these diseases during the health emergency, and strengthens surveillance systems, correcting weaknesses found throughout the pandemic. The indirect effects of the pandemic are essential information to guide public health policies that ensure resilient responses from the post-pandemic health system, or in new emergency situations that may arise.

The study was carried out aiming at evaluating the indirect impact of the COVID-19 pandemic on notifiable disease control programs, using as an example the comparison of the national and state situations, and the case of a medium-sized municipality that before COVID-19 had an organized public health system, with adequate coverage and consolidated experience in Primary Health Care. The temporal analysis was supported by incidence or detection rates for notified and confirmed cases of dengue, tuberculosis, congenital and gestational syphilis, according to the place of occurrence, subdivided into two periods - before and during the COVID-19 pandemic. These rates can highlight the weaknesses of official figures and help decision-makers evaluate new policies and measures for health interventions.

## METHOD

### Design of Study

Epidemiological, ecological, time series study.

### Context (Local, Population and Data Collection)

Notified and confirmed cases of tuberculosis, congenital syphilis, gestational syphilis and dengue fever in the municipality of Ponta Grossa, in the state of Paraná and in Brazil were studied. The data refer to the years 2015–2019 (pre-COVID-19 pandemic period) and 2020–2021 (first two years of the COVID-19 pandemic). Ponta Grossa is a medium-sized municipality in the State of Paraná, located in the southern region of Brazil. In January/2020, the Municipal Health Foundation of Ponta Grossa reported 78 Family Health Strategy teams, with 78.96% coverage of Primary Care (277,740 inhabitants served)^(4)^.

Data collection was carried out between 2023 and 2024. To ensure data quality and nominal confidentiality of cases, and to reduce bias, data extraction was carried out by the Municipal Epidemiology Coordination and the Specialized Assistance Service/Testing and Counseling Center (*SAE/CT*A), under the supervision of a researcher participating in the study. The data were extracted by the Tabulator for Windows (TABWIN) of the *SUS* Information Technology Department (*DATASUS*) and converted into Excel spreadsheets.

Data were filtered by year of diagnosis, municipality of residence, final classification, or closure status. Cases of recurrence, readmission, and transfer of tuberculosis were excluded from the analyzed database. For tuberculosis, only pulmonary cases were included, or pulmonary and extrapulmonary cases when diagnosed together. The Brazilian Notifiable Diseases Information System (*SINAN*) confirmation classification was used for each disease, arranged in the data dictionaries of the notification forms.

### Variables

The following exposure variables were considered: year and place of occurrence, age group and biological sex. The outcome variables were the notified and confirmed cases of the notifiable diseases studied, and the incidence rates (gross and standardized) of tuberculosis, dengue, gestational syphilis and congenital syphilis.

### Data Sources

The municipal data were extracted from *SINAN* at the Municipal Epidemiology Coordination. State and national information on live births and confirmed cases of diseases were collected by the DATASUS “TABNET” tabulation tool^(5)^. Collections on diseases refer to data coming primarily from compulsory notifications.

### Statistical Methods

Qualitative variables were described in simple and relative frequencies, aiming at evaluating the notified and confirmed cases by year, sex, and age group. Gross and standardized incidence/detection rates were calculated. The gross municipal rates were very close to the standardized ones, so it was decided that the differences in averages and temporal trends would be evaluated with the standardized rates for state and national comparison purposes. The standardized incidence rates, for reported/confirmed cases, were calculated using the population of the state of Paraná from 2015 to 2021 as the standard population. Dengue and tuberculosis rates were standardized as follows: Standardized rate = 100000 *((Standard Population × (Cases/Population))/(Population + Standard Population)). This formula was similarly used for divisions by sex and age group. The standardization of gestational and congenital syphilis rates was done as follows: Expected cases = Gross rate × Standard live births (Live births in the state of Paraná); Standardized rate = (Expected cases/(Live births + Standard live births)) × 1000. The averages and standard deviations of the standardized rates for the periods 2015–2019 and 2020–2021 are calculated, as well as the percentage variation between the average rates.

To assess temporal trends, linear regression models were developed, with standardized rates as the dependent variable and the year as the independent variable. The coefficient (β) and its confidence intervals (95%CI), the coefficient of determination (R^2^), and the p-value of the normality test of the residues were evaluated as a diagnosis of model adjustment. In assessing the differences between the average rates in the pre-pandemic (2015–2019) and pandemic (2020–2021) periods, Student’s t-tests were performed, when there was adherence of data to normal distribution (gross and standardized rates of notified cases of Dengue, Tuberculosis and Gestational Syphilis, and gross and standardized rates of confirmed cases of Tuberculosis, Congenital and Gestational Syphilis), or Mann-Whitney U test, when there was no normality for the data (gross and standardized rates of notified cases of Congenital Syphilis and confirmed cases of dengue). The significance level used was 5% and all analyses were performed in the R 4.1.0 environment^(6)^. The *Strengthening the Reporting of Observational Studies in Epidemiology* (STROBE) guidelines were followed to conduct the study.

### Ethical Aspects

This study was part of the research “COVID-19 Pandemic: characteristics and responses of the Brazilian Public Health System and surveillance of long-COVID in a medium-sized municipality”, approved by the Ethics Committee of the Universidade Estadual de Ponta Grossa, protocol 5.318.200/2022, and is in compliance with Resolution 466/12.

## RESULTS

During the period analyzed, 969 cases of dengue, 510 of tuberculosis, 318 of congenital syphilis, and 779 of gestational syphilis were reported in Ponta Grossa. Of these, 144 (14.86%), 510 (100.0%), 154 (48.42%), and 771 (98.97%) were confirmed for dengue, tuberculosis, congenital and gestational syphilis, respectively. In 2016, there was the highest percentage of dengue notifications (28.59%). More than 40% of notified cases, and 32% of confirmed cases of congenital syphilis occurred in 2021; 41% of all confirmed cases of gestational syphilis in the period studied occurred in the first two years of the COVID-19 pandemic (Table S1).

For confirmed cases, gross dengue incidence rates were higher in 2016 and 2020. From 2019 onwards, the incidence of tuberculosis increased between 4 and 12 new cases compared to previous years. Comparing the first and last years of the historical series, it can be seen that the gross incidence rate of congenital syphilis and the detection rate of gestational syphilis increased by 9 and 26 new cases, respectively (Table 1).

**Table 1:** Gross incidence or detection (100 thousand inhabitants) / (1000 live births) rates for notified and confirmed cases of dengue, tuberculosis, congenital and gestational syphilis, according to year of occurrence - Ponta Grossa, PR, 2015–2021.

Cases	Disease	2015	2016	2017	2018	2019	2020	2021	2015–2019		2020–2021		p	Range (%)
		Rate	Rate	Rate	Rate	Rate	Rate	Rate	M	SD	M	SD		
Notified	Dengue*	19.830	81.201	8.713	5.746	46.057	69.230	46.539	32.31	31.61	57.88	16.05	0.230^1^	79.14
	Tuberculosis*	14.503	18.175	12.778	19.825	25.587	25.891	28.982	18.07	5.01	27.44	2.19	0.022^1^	51.85
	Congenital Syphilis**	1.302	1.311	3.023	2.216	5.005	25.327	27.713	2.57	1.57	26.52	1.69	0.095^2^	931.91
	Gestational Syphilis**	11.721	18.165	20.975	15.328	19.219	29.970	39.560	17.08	3.63	34.77	6.78	0.138^1^	103.57
Confirmed	Dengue*	1.776	15.243	0.000	0.287	4.833	14.915	4.180	4.43	6.34	9.55	7.59	0.571^2^	115.58
	Tuberculosis*	14.503	18.175	12.778	19.825	25.587	25.891	28.982	18.07	5.01	27.44	2.19	0.022^1^	51.85
	Congenital Syphilis**	1.302	1.124	3.023	2.031	4.805	8.442	10.578	2.46	1.51	9.51	1.51	0.035^1^	286.59
	Gestational Syphilis**	11.721	18.165	20.975	15.328	19.019	29.548	38.502	17.04	3.60	34.03	6.33	0.131^1^	99.71

*Rate = Gross incidence rate (100,000 inhab.) for reported cases;

**Gross incidence rate (1,000 live births) for notified cases; M = average rate for the period; SD = standard deviation;

^1^Student’s t-test;

^2^Mann-Whitney U-Test.

Comparing the periods from 2015–2019 and 2020–2021, there was an increase in the standardized average rate of all diseases analyzed in the first two years of the COVID-19 pandemic (Table 2). Tuberculosis was the disease with the smallest increase in the average number of reported and confirmed cases (59.06%). On average, before the COVID-19 pandemic, for every 1000 live births, 2.38 cases of congenital syphilis and 16.49 of gestational syphilis were confirmed. These average rates rose to 9.21 and 32.94, respectively. There was a variation of 931% for notification of congenital syphilis. For confirmed cases, the variation was 286% (Table 2). There was a statistically significant difference in the average rates of confirmed cases of tuberculosis and congenital syphilis between the periods of 2015–2019/2020–2021. However, when analyzing by age group, the differences in the average rates of confirmed cases remained for tuberculosis among the older people (Table S2).

**Table 2:** Standardized incidence or detection (100 thousand inhabitants) / (1000 live births) rates for notified and confirmed cases of dengue, tuberculosis, congenital and gestational syphilis, according to year, and comparison between periods. Ponta Grossa, Paraná: 2015–2021.

Cases	Disease	2015	2016	2017	2018	2019	2020	2021	2015–2019		2020–2021		p	Range (%)
		Rate	Rate	Rate	Rate	Rate	Rate	Rate	M	SD	M	SD		
Notified	Dengue^*^	19.244	78.796	8.454	5.575	44.683	67.158	45.142	31.35	30.68	56.15	15.57	0.230^1^	79.11
	Tuberculosis^*^	14.074	17.637	12.399	19.235	24.824	25.116	28.113	16.73	4.86	26.61	2.12	0.022^1^	59.06
	Congenital Syphilis^**^	1.260	1.267	2.925	2.142	4.847	24.533	26.820	2.49	1.49	25.68	1.62	0.095^2^	931.33
	Gestational Syphilis^**^	11.342	17.560	20.294	14.814	18.613	29.030	38.285	16.52	3.52	33.66	6.54	0.138^1^	103.75
Confirmed	Dengue^*^	1.723	14.792	0.000	0.279	4.689	14.469	4.055	4.30	6.15	9.26	7.36	0.571^2^	115.35
	Tuberculosis^*^	14.074	17.637	12.399	19.235	24.824	25.116	28.113	16.73	4.86	26.61	2.12	0.022^1^	59.06
	Congenital Syphilis^**^	1.260	1.086	2.925	1.963	4.653	8.178	10.237	2.38	1.46	9.21	1.46	0.034^1^	286.97
	Gestational Syphilis^**^	11.342	17.560	20.294	14.814	18.420	28.621	37.262	16.49	3.49	32.94	6.11	0.130^1^	99.76

*Rate = Standardized incidence rate (100,000 inhab.) for reported cases with the state of Paraná as the standard population;

**Standardized incidence rate (1,000 live births) for notified cases with live births in the state of Paraná as the standard population, M = average rate for the period; SD = standard deviation;

^1^Student’s t-test;

^2^Mann-Whitney U-Test.

In regression analysis, an increasing trend of notifications (β = 4.47, p = 0.016) of congenital syphilis was observed between 2015 and 2021 (Table 3). There was an increase in the detection rate of gestational syphilis considering all notified and confirmed cases. In pregnant women aged between 15–19 and 20–29, for each year passed, the detection of confirmed cases increased by 6.96 (p = 0.001) and 4.31 (p = 0.019) cases/1000 LB, respectively.

**Table 3:** Linear regression model having as dependent variable the standardized incidence rate of reported/confirmed cases of congenital syphilis, gestational syphilis (100 thousand live births) and tuberculosis (100 thousand inhabitants), and as independent variable the year, according to sex and age group - Ponta Grossa-PR: 2015–2021.

Model	β	CI 95%	P value	R^2^	Normality of residues*
**Congenital syphilis**					
*Notified cases*					
All	4.47	1.24 – 7.69	0.016	0.72	0.365
Female Sex	4.66	1.36 – 7.96	0.015	0.73	0.164
Male sex	4.31	1.31 – 7.32	0.014	0.73	0.623
*Confirmed cases*					
All	1.53	0.78 – 2.28	0.003	0.85	0.625
Female Sex	1.46	0.62 – 2.31	0.007	0.80	0.628
Male sex	1.65	0.75 – 2.55	0.005	0.82	0.734
**Gestational syphilis**					
*Notified cases*					
All	3.65	1.08 – 6.21	0.015	0.73	0.257
Age group (years)			
10 – 14	4.94	–10.15 – 20.03	0.438	0.12	0.951
15 – 19	7.43	4.73 – 10.12	0.001	0.91	0.387
20 – 29	4.74	0.91 – 8.57	0.025	0.67	0.288
30 – 39	1.99	–0.02 – 3.99	0.051	0.56	0.148
40 – 49	1.54	–2.74 – 5.82	0.397	0.15	0.040
*Confirmed cases*					
All	3.50	1.03 – 5.98	0.015	0.73	0.217
Age group (years)					
10 – 14	4.58	–14.97 – 24.14	0.573	0.07	0.744
15 – 19	6.96	4.39 – 9.53	0.001	0.91	0.519
20 – 29	4.31	1.06 – 7.57	0.019	0.70	0.591
30 – 39	1.73	0.15 – 3.31	0.038	0.61	0.093
40 – 49	1.98	–2.81 – 6.77	0.336	0.18	0.396
**Tuberculosis**				
*Notified and confirmed cases*					
All	2.48	1.08 – 3.88	0.006	0.81	0.125
Female Sex	1.29	0.23 – 2.35	0.026	0.66	0.564
Male sex	3.64	0.79 – 6.49	0.022	0.68	0.759
Age group (years)					
0 – 4	–0.40	–1.00 – 0.20	0.144	0.38	0.803
5 – 9	0.26	–0.42 – 0.94	0.363	0.17	0.098
10 – 14	<0.01	–0.75 – 0.75	1.000	<0.01	<0.001
15 – 19	–0.60	–3.99 – 2.79	0.666	0.04	0.366
20 – 29	5.19	1.13 – 9.24	0.022	0.68	0.219
30 – 39	3.86	–0.32 – 8.04	0.064	0.53	0.660
40 – 49	2.81	–0.53 – 6.15	0.083	0.48	0.076
50 – 59	1.88	0.36 – 3.40	0.025	0.67	0.420
60 – 69	3.67	–1.13 – 8.47	0.107	0.44	0.985
70 – 79	6.45	–0.49 – 13.38	0.062	0.53	0.977
80 +	2.63	–5.76 – 11.02	0.457	0.11	0.388

β = model coefficient; R^2^= coefficient of determination;

*p–value of the Shapiro-Wilk test.

Dengue fever did not show an upward or downward trend in any of the situations tested. The incidence of tuberculosis increased during the 7 years of follow-up. Analyzing the historical trend of tuberculosis by age group, it was found that the growth occurred among young adults (Table 3).

**Figure 1:**
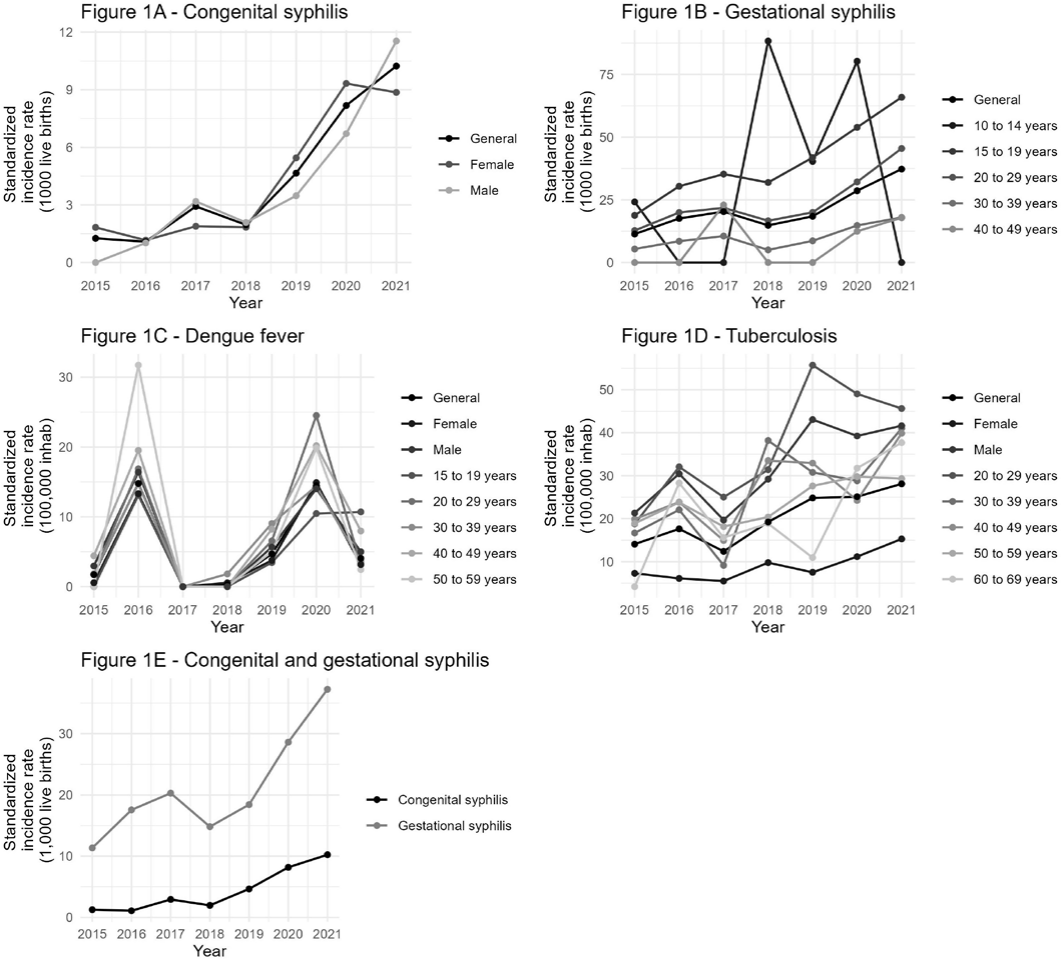
Standardized incidence or detection rates (1000 live births) for confirmed cases of congenital syphilis, gestational syphilis, dengue and tuberculosis (100 thousand inhabitants), according to year of occurrence, sex or age group. Ponta Grossa-PR: 2015–2021.

Figure 1 summarizes the behavior of the incidence of confirmed cases of the diseases studied, from 2015 to 2021, in Ponta Grossa.

Comparing the standardized incidence/detection rates among Brazil, Paraná and Ponta Grossa, dengue had a national reduction greater than 30% in average incidence rates in 2021–2021 versus 2015–2019, while Paraná grew 355% and Ponta Grossa 115% on average. Regarding tuberculosis, the municipal incidence rate was one and a half times higher than the state rate in 2015 and 7 times higher than the national rate. However, in 2021, Ponta Grossa had a rate 3 times higher than the state and 14 times higher than the country (Table S3).

Regarding congenital syphilis, the country began 2015 with a confirmed case rate of 0.33 and ended 2021 with 0.20/1000 LB. Paraná also saw a drop in the period with a negative variation of 25.49% comparing 2020–2021 with 2015–2019. In contrast, Ponta Grossa started 2015 with 1.26 and ended 2021 with an incidence rate of 10.23 new cases/1000 LB. Regarding gestational syphilis, in 2015 the detection rate in Ponta Grossa was twice that of Paraná and 20 times lower than that of Brazil. However, in 2021 the national, state and municipal rates were, respectively, 0.5, 3.8 and 37/1000 LB (Table S3). The differences in means 2015–2019/2020–2021 were statistically significant only for tuberculosis at the municipal level.

**Figure 2:**
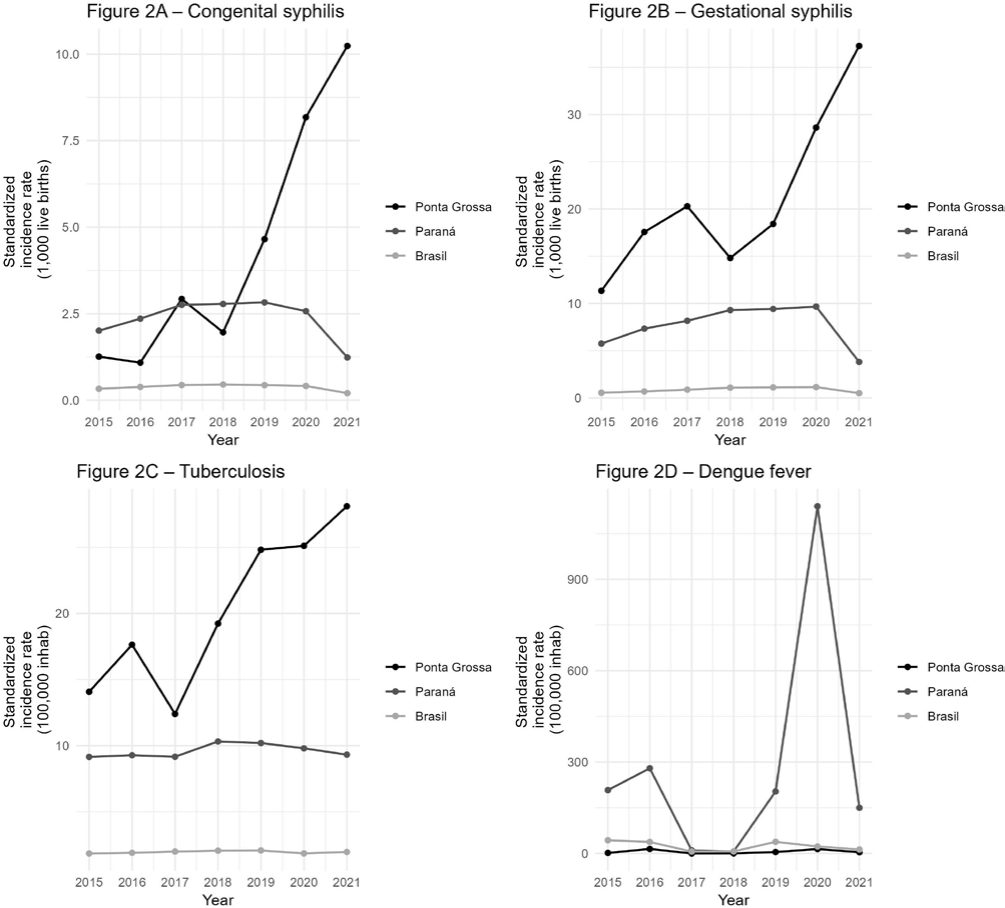
Standardized incidence or detection rates for confirmed cases of congenital syphilis, gestational syphilis (1,000 live births), dengue fever, and tuberculosis (100,000 inhabitants), in Brazil, Paraná and Ponta Grossa between 2015 and 2021. β (p-value) Congenital syphilis: Ponta Grossa = 1.53(0.003); Paraná = −0.06(0.598); Brazil = −0.01(0.538). β (p-value) Gestational syphilis: Ponta Grossa = 3.50(0.015); Paraná = <0.01(0.994); Brazil = 0.04(0.530). β (p-value) Tuberculosis: Ponta Grossa = 2.48 (0.006); Paraná = 0.09(0.373); Brazil = 0.01(0.533). β (p-value) Dengue: Ponta Grossa = 0.39(0.774); Paraná = 62.14(0.450); Brazil = −3.18(0.333).

Figure 2 shows comparisons of standardized rates for the diseases studied between country, state and municipality. The trends were increasing and statistically significant only for the municipality of Ponta Grossa and for the diseases congenital syphilis (p = 0.003), gestational syphilis (p = 0.015), and tuberculosis (p = 0.006).

## DISCUSSION

The main results of this study show that, in the first two years of the pandemic, there was an indirect impact of COVID-19 on the control of tuberculosis and gestational and congenital syphilis in the municipality studied. The average incidence rate for these 3 diseases was higher in the first two pandemic years, when compared to the previous period. Municipal rates did not keep up with state and national reductions and were higher during the pandemic. Over the seven years analyzed, there was a tendency for the incidence rate of tuberculosis and gestational syphilis to increase at the municipal level.

The study has the limitation of having been planned to verify the effect of the pandemic on the SUS response in the first two pandemic years. Therefore, it was not possible to visualize the effect of the recovery of health services after the reduction in morbidity and mortality due to COVID-19 and the end of the emergency in the following years. Despite this, the first years of the pandemic tell the story of the flexibility and resilience of surveillance systems and health services. Another limitation is the difficulty in comparing the data with information from the epidemiological bulletins of the Ministry of Health and the State of Paraná because these are not presented with standardized rates. There is a limitation in the design of the study of municipal, state, and national aggregate data. Therefore, the results do not represent specific health units or populations. It is also considered that social isolation during the pandemic period favored cases underreporting. Finally, manually filling of data on notification forms and subsequent transcription to information systems can result in integrity problems or loss of information.

Tuberculosis and syphilis diagnoses are generally decentralized to Primary Health Units in Brazilian territories, including Ponta Grossa. During the COVID-19 pandemic, there was a collective effort to maintain Primary Care services, especially prenatal care and care for people with chronic conditions. However, lack of knowledge about COVID-19 and the lack of immunization in the first year of the pandemic promoted fear among the population and pregnant women stopped carrying out prenatal care properly and the frequency of prenatal exams was reduced^(7,8)^. Women have been overwhelmed by social distancing^(9)^. Transport was no longer offered regularly during certain periods, families became poorer^(10)^, there was domestic violence^(9,11)^, direct contact with infected people for long periods. The exponential increase in information during the pandemic (infodemic), especially fake news, were seen by health professionals as an obstacle in the fight against COVID-19^(12)^. Many Brazilians receiving healthcare services believed the fake news, hindering positive healthcare results. Added to this are the difficulties in organizing healthcare teams in the face of the immense challenges of COVID-19.

Such factors may have reflected in the growth of the diseases studied. Consequences as hospitalizations and maternal mortality during the pandemic have been documented^(13,14)^, and an increase in deaths from severe acute respiratory syndrome among pregnant women was observed in Brazil and Paraná, associated with the end of pregnancy, cardiovascular conditions, and age over 35 years. Mortality in children under 5 years of age decreased in 2020 in Brazil, compared to 2017. However, many deaths occurred in the early neonatal period^(15)^. Conditions originating in the perinatal period were the most frequent causes of child deaths in 2020, followed by congenital malformations, diseases of the respiratory system, external causes, and infectious and parasitic diseases^(15)^. In the present study, an increase in notifications and confirmations of syphilis was observed for the mother-child binomial, a finding that could be associated with the groups of main causes of children death during the pandemic.

The gross rates presented for the municipality are consistent with the official information^(16)^. The national and state congenital and gestational syphilis rates presented in this study differ from those in the Ministry of Health’s Epidemiological Bulletin^(17)^. The authors chose to present standardized rates of confirmed cases, while national data are described with gross rates and notified cases. Comparability between standardized rates excludes the effect of population size and composition. The presentation of confirmed cases excludes the possibility of overreporting.

Among the diseases evaluated, dengue was the most reported and the least confirmed in the period analyzed. Dengue is a disease that has nonspecific initial clinical presentations and requires differential diagnoses for exanthematous, febrile, meningeal, and other clinical syndromes. Dengue fever is underreported in Brazil^(18)^, and may not be suspected, or have the notification opened and the case discarded after the laboratory tests results. Autochthonous cases of dengue fever in the municipality studied were diagnosed for the first time in 2016, and the difference between the high notification and confirmation rates could suggest that teams are learning to recognize the disease. The territory of Ponta Grossa is located in the southern region, a place with lower average temperatures. An increase in cases in the city was expected because the dengue vector spread throughout the country, but there was no significant growth trend in the period analyzed. It is likely that confirmed cases have not increased as in other Brazilian regions due to the environmental and social conditions of the region, or due to the low specificity of the surveillance system as reported in the literature^(18)^. In contrast, in the early stages of the disease, COVID-19 and dengue have similar clinical manifestations and laboratory characteristics and this situation may have contributed to the increase in dengue notifications in Ponta Grossa during the pandemic, especially in 2020, when there were not enough tests for COVID-19^(19)^.

Comparing 2022 with the previous year, authors found a significant increase in dengue cases in Brazil^(20)^. After the first two years of the pandemic, the dengue epidemic reached regions that had not previously had outbreaks, such as the south of the country. The hypothesis of substitution of dominance from DENV2 to DENV1 serotype may have contributed to the explosion of cases in Brazil observed from 2022 onwards. According to the authors^(20)^, in the early years of COVID-19, there was underreporting of all other diseases because of the decline in non-COVID medical care, closure of non-essential services, and measures that limited spatial mobility. These authors’ comments emphasize that the lower national dengue incidence rates during the pandemic represent the indirect impact of the measures adopted to control COVID-19. The fact that no growth trend of dengue fever was observed in Ponta Grossa, between 2015 and 2021, could be explained in part by the work process in the services, low susceptibility of the population to the circulating serotype and the local socioeconomic conditions that protected against the proliferation of the mosquito. However, the historical series must be monitored in the years following COVID-19, because the municipality may suffer the effect of post-pandemic social determinants that favor the proliferation of the mosquito, added to the unexpected periods of heavy rains and the change in the predominant viral serotype in the country.

The proportion of notified and confirmed tuberculosis cases was similar because cases are reported to the surveillance system only when confirmed by clinical, epidemiological, and/or laboratory criteria, preferably by the rapid PCR test. Gestational and congenital syphilis had high confirmation rates, suggesting that the surveillance system has implemented strategies that increase the sensitivity of notifications and the positive predictive value. Although confirmations of congenital syphilis increased in 2020 and 2021, there was a proportional reduction in confirmations in relation to notifications, when compared to previous years. Less availability of laboratory diagnostics during the pandemic could be a plausible explanation for the time frame, but clinical-laboratory experience may have grown to complete diagnoses, and could also be an indication that the provision of treatment to mothers during prenatal care was showing signs of effectiveness. It should be noted that the gross and standardized rates of congenital syphilis are very high and far from the WHO target of less than 0.5 cases/1000 LB for the elimination of vertical transmission. It is assumed then that the target of 95% treatment of pregnant women with syphilis had not yet been achieved^(17)^.

The average incidence rates of gestational syphilis showed significant differences between adolescent and young adult women, both for cases notifications and confirmations. Trend analysis showed that there was an increase in new cases for adolescents and young women. The increase in gestational syphilis in these age groups suggests, among other things, unprotected sexual initiation, vulnerability of women of reproductive age, and lack of commitment by the partner to the prevention and treatment of syphilis during pregnancy. The increasing incidence of gestational syphilis was followed by an increase in the incidence of congenital syphilis, indicating the vulnerability of the fetus to the practices of parents, the public policies adopted, and the behavior of society in the face of a disease that is well recognized scientifically, but still far from being resolved socially. Rates of congenital syphilis lower than rates of gestational syphilis reveal that mothers are being treated throughout pregnancy and the binomial is being protected from sequelae.

The average number of confirmed tuberculosis cases increased during the COVID-19 pandemic among older people. The incidence growing trend of tuberculosis between 2015 and 2021 was observed in young adults. Although it was not the subject of this study, the literature suggests that the incidence of tuberculosis associated with HIV co-infection has increased among young people. In a national cohort, over 64% of people living with HIV were aged 20–39 years, and 6% had tuberculosis^(21)^. The international literature reveals a percentage close to the national one, with 9% of young adults having tuberculosis^(22)^.

Conversely, in the pandemic years, the highest rate of hospital admissions for SARS occurred among adults and older people. It is likely that the increase in lung imaging and differential diagnoses for COVID-19 have increased the parallel diagnosis for tuberculosis. From 2018 onwards, the incidence rate for tuberculosis grew rapidly in Ponta Grossa, fell slightly in 2020, and rose again in 2021. This pattern was very different from the state and in Brazil, which showed a constant and downward trend. Descriptively analyzing the impact of the COVID-19 pandemic on pulmonary tuberculosis, authors^(3)^ raised concerns that the pandemic would make elimination goals more difficult. Treatment abandonment, combined with restrictions on services, resources and supplies, led to a drop in the number of confirmed cases in all Brazilian regions, except the North.

Brazil has participated in national and international strategies to reduce tuberculosis, and Paraná has followed these public policies. However, faster, more sensitive and specific tests for tuberculosis can contribute to higher incidences in specific territories. In 2018, the municipality of Ponta Grossa was awarded a GeneXpert® device by the State Health Department, through a federal surveillance program, and joined the state network for the diagnosis of tuberculosis. The introduction of this equipment represented a substantial change in the disease surveillance profile, increasing the sensitivity of sample analysis. Before this initiative, samples were subjected to the acid-fast bacillus (AFB) methodology, which presents significant limitations for diagnosis. From this inflection point onwards, municipal flows were restructured, making the rapid molecular test for tuberculosis the first option for diagnosing new cases in all municipal services. This transition resulted in a significant improvement in the early detection of tuberculosis and allowed faster and more accurate diagnosis. As rapid molecular tests for tuberculosis are not yet available to all Brazilians with respiratory symptoms, the question remains as to whether the findings in Ponta Grossa correspond to an improvement in the sensitivity and predictive value of the surveillance system, when compared to the rest of the state and country, or whether the number of new cases has increased due to the increase in transmission of the disease.

## CONCLUSION

COVID-19 indirectly impacted the control of tuberculosis, congenital and gestational syphilis in the municipality studied, promoting higher average incidence rates in the first years of the pandemic. The temporal trends were increasing for the municipality, but the high rates did not follow the national and state trends.

The findings of this research suggest that health surveillance should be municipalized for local priorities. Furthermore, they support local management in assessing the health situation, verifying the consistency and potential of the surveillance system, and promoting ongoing strategies to reduce the incidence of tuberculosis, syphilis, and dengue fever. The results confirm, for this and other medium-sized municipalities, that public health emergencies are powerful in disrupting the routine of services and simultaneously increasing the incidence of other health problems. Therefore, municipal assessments and monitoring are relevant to the SUS and must be carried out continuously before emergencies occur. Following international health regulations and national recommendations, municipalities must develop plans to prevent and respond to public health emergencies. Epidemiological surveillance in this process should guide public policies. It is suggested that cross-sectional and cohort epidemiological studies can be conducted on notifiable diseases so that the determining factors for the incidence of the diseases studied can be understood.

## Data Availability

The research data were deposited in the SciELO Data repository and can be accessed at the link: https://doi.org/10.48331/scielodata.LTXYKD.

## SUPPLEMENTARY MATERIAL

The following online material is available for this article:

Table S1 – Number and Percentage of Notified and Confirmed Cases of Dengue, Tuberculosis, Congenital and Gestational Syphilis, by Year of Occurrence. Ponta Grossa, Paraná, Brazil: 2015–2021.

Table S2 – Standardized incidence rates (100 thousand inhabitants)/(1000 live births) confirmed cases of dengue, tuberculosis, congenital, and gestational syphilis by age group, according to years and period comparison. Ponta Grossa, Brazil: 2015–2021.

Table S3 – Standardized incidence rates (100 thousands inhabitants)/(1000 live births) for confirmed cases of dengue, tuberculosis, congenital, and gestational syphilis, by year and period comparison. Brazil, Paraná, and Ponta Grossa: 2015–2021.
