# Supplementary material for: Impact of COVID-19 on notifiable diseases: a time series study
Source: Rev Esc Enferm USP. 2025 Feb 17;58:e20240098. doi: 10.1590/1980-220X-REEUSP-2024-0098en (PMC11884405; doi:10.1590/1980-220X-REEUSP-2024-0098en)
Supplement: Supplementary file 1 [file 1980-220X-reeusp-58-e20240098-Table-S1.pdf]

Rev Esc Enferm USP
https://doi.org/10.1590/1980-220X-REEUSP-2024-0098en

Supplementary Material to "Impact of COVID-19 on notifiable diseases: a time series study"

Table S1 - Number and Percentage of Notified and Confirmed Cases of Dengue, Tuberculosis, Congenital and Gestational Syphilis, by Year of Occurrence. Ponta Grossa, Paraná, Brazil: 2015–2021.

		**2015**		**2016**		**2017**		**2018**		**2019**		**2020**		**2021**	
**Casos**	**Doença**	**N**	**%**	**N**	**%**	**N**	**%**	**N**	**%**	**N**	**%**	**N**	**%**	**N**	**%**
Notified	Dengue	67	6,91	277	28,59	30	3,1	20	2,06	162	16,72	246	25,39	167	17,23
	Tuberculosis	49	9,61	62	12,16	44	8,63	69	13,53	90	17,65	92	18,04	104	20,39
	Congenital syphilis	7	2,2	7	2,2	16	5,03	12	3,77	25	7,86	120	37,74	131	41,19
	Gestational syphilis	63	8,09	97	12,45	111	14,25	83	10,65	96	12,32	142	18,23	187	24,01
Confirmed	Dengue	6	4,17	52	36,11	0	0	1	0,69	17	11,81	53	36,81	15	10,42
	Tuberculosis	49	9,61	62	12,16	44	8,63	69	13,53	90	17,65	92	18,04	104	20,39
	Congenital syphilis	7	4,55	6	3,9	16	10,39	11	7,14	24	15,58	40	25,97	50	32,47
	Gestational syphilis	63	8,17	97	12,58	111	14,4	83	10,77	95	12,32	140	18,16	182	23,61

N = Number of notified or confirmed cases; % = Percentage of notified/confirmed cases in relation to the total number of cases across all years.
