# Supplementary material for: Impact of COVID-19 on notifiable diseases: a time series study
Source: Rev Esc Enferm USP. 2025 Feb 17;58:e20240098. doi: 10.1590/1980-220X-REEUSP-2024-0098en (PMC11884405; doi:10.1590/1980-220X-REEUSP-2024-0098en)
Supplement: Supplementary file 2 [file 1980-220X-reeusp-58-e20240098-Table-S2.pdf]

Rev Esc Enferm USP
https://doi.org/10.1590/1980-220X-REEUSP-2024-0098en

Supplementary Material to "Impact of COVID-19 on notifiable diseases: a time series study"

Table S2 – Standardized incidence rates (100 thousand inhabitants) / (1000 live births) confirmed cases of dengue, tuberculosis, congenital, and gestational syphilis by age group, according to years and period comparison. Ponta Grossa, Brazil: 2015–2021.

**Disease (Confirmed cases)**		**2015**	**2016**	**2017**	**2018**	**2019**	**2020**	**2021**	**2015-2019**		**2020-2021**		**p**	**Range (%)**
		**Rate**	**Rate**	**Rate**	**Rate**	**Rate**	**Rate**	**Rate**	**M**	**DP**	**M**	**DP**		
	Age group (years)													
Dengue *	15 - 19	0.000	13.097	0.000	0.000	3.448	10.471	10.707	3,31	5,67	10,59	0,17	0,045	219,94
	20 - 29	1.708	16.874	0.000	0.000	6.556	24.518	3.260	5,03	7,15	13,89	15,03	0,553	176,14
	30 - 39	1.853	14.714	0.000	1.819	9.050	14.412	3.565	5,49	6,22	8,99	7,67	0,637	63,75
	40 - 49	4.418	19.526	0.000	0.000	8.232	20.199	7.974	6,44	8,09	14,09	8,64	0,406	118,79
	50 - 59	0.000	31.737	0.000	0.000	5.023	19.859	2.443	7,35	13,80	11,15	12,32	0,547	51,70
Tuberculosis*	0 - 4	3.763	0.000	0.000	0.000	0.000	0.000	0.000	0,75	1,68	0,00	0,00	0,752	-100,00
	5 - 9	0.000	0.000	0.000	0.000	0.000	3.700	0.000	0,00	0,00	1,85	2,62	0,206	-
	10 - 14	0.000	0.000	0.000	3.740	0.000	0.000	0.000	0,75	1,67	0,00	0,00	0,752	-100,00
	15 - 19	13.033	9.822	3.318	3.382	17.242	13.962	0.000	9,36	6,08	6,98	9,87	0,794	-25,43
	20 - 29	18.785	32.060	25.026	31.379	55.722	49.036	45.634	32,59	14,01	47,34	2,41	0,078	45,26
	30 - 39	16.674	22.071	9.141	38.191	30.770	28.824	40.999	23,37	11,44	34,91	8,61	0,256	49,38
	40 - 49	19.882	23.865	14.924	33.520	32.927	24.239	39.872	25,02	8,13	32,06	11,05	0,526	28,14
	**50 - 59**	**18.899**	**23.803**	**18.159**	**20.388**	**27.629**	**29.789**	**29.321**	**21,78**	**3,93**	**29,56**	**0,33**	**0,011**	**35,72**
	**60 - 69**	**4.194**	**28.301**	**15.614**	**18.859**	**10.943**	**31.752**	**37.686**	**15,58**	**9,00**	**34,72**	**4,20**	**0,016**	**122,85**
	**70 - 79**	**25.514**	**8.152**	**7.808**	**7.471**	**42.851**	**40.923**	**52.150**	**18,36**	**15,69**	**46,54**	**7,94**	**0,034**	**153,49**
	80 +	0.000	35.329	0.000	0.000	0.000	29.562	28.375	7,07	15,80	28,97	0,84	0,285	309,76
Gestacional syphilis **	10 - 14	24.173	0.000	0.000	88.322	40.312	80.271	0.000	23,14	21,64	40,14	56,76	0,746	73,47%
	15 - 19	18.810	30.413	35.266	31.900	41.855	53.911	65.891	30,40	8,40	60,82	9,77	0,082	100,07%
	20 - 29	12.752	19.951	21.814	16.674	19.999	32.153	45.476	17,58	5,32	40,22	9,71	0,156	128,78%
	30 - 39	5.399	8.501	10.565	5.035	8.645	14.772	17.987	7,29	2,94	17,59	3,11	0,068	141,29%
	40 - 49	0.000	0.000	22.954	0.000	0.000	12.442	17.845	5,71	9,94	12,17	0,39	0,220	113,13%

Rate = *Standardized incidence rate (100 thousand inhabitants) for confirmed cases with population of the state of Paraná as the standard/**Standardized incidence rate (1000 live births) for confirmed cases using live births in the state of Paraná as the standard population.
