# Supplementary material for: Impact of COVID-19 on notifiable diseases: a time series study
Source: Rev Esc Enferm USP. 2025 Feb 17;58:e20240098. doi: 10.1590/1980-220X-REEUSP-2024-0098en (PMC11884405; doi:10.1590/1980-220X-REEUSP-2024-0098en)
Supplement: Supplementary file 3 [file 1980-220X-reeusp-58-e20240098-Table-S3.pdf]

Rev Esc Enferm USP
https://doi.org/10.1590/1980-220X-REEUSP-2024-0098en

Supplementary Material to "Impact of COVID-19 on notifiable diseases: a time series study"

Table S3 – Standardized incidence rates (100 thousands inhabitants)/(1000 live births) for confirmed cases of dengue, tuberculosis, congenital, and gestational syphilis, by year and period comparison. Brazil, Paraná, and Ponta Grossa: 2015–2021.

**Doença (confirmed cases)**		**2015**	**2016**	**2017**	**2018**	**2019**	**2020**	**2021**	**2015-2019**		**2020-2021**		**p**	**Range (%)**
		**Rate**	**Rate**	**Rate**	**Rate**	**Rate**	**Rate**	**Rate**	**M**	**DP**	**M**	**DP**		
Dengue*	Brazil	43,394	37,719	6,040	6,667	38,146	23,007	12,858	26,39	18,43	17,93	7,18	0,423	-32,06
	Paraná	207,917	279,491	9,825	6,397	203,565	1139,779	149830,	141,44	125,40	644,80	700,00	0,571	355,88
	Ponta Grossa	1,723	14,792	0,000	0,279	4,689	14,469	4,055	4,30	6,15	9,26	7,36	0,571	115,35
Tuberculosis*	Brazil	1,841	1,887	1,982	2,049	2,065	1,847	1,954	1,96	0,10	1,90	0,08	0,431	-3,06
	Paraná	9,149	9,270	9,159	10,314	10,198	9,794	9,317	9,62	0,59	9,56	0,34	0,870	-0,62
	Ponta Grossa	14,074	17,637	12,399	19,235	24,824	25,116	28,113	17,63	4,86	26,61	2,12	0,022	50,94
Congenital syphilis**	Brazil	0,331	0,384	0,438	0,454	0,437	0,412	0,205	0,41	0,05	0,31	0,15	0,071	-24,39
	Paraná	2,010	2,357	2,758	2,782	2,828	2,574	1,236	2,55	0,35	1,90	0,95	0,508	-25,49
	Ponta Grossa	1,260	1,086	2,925	1,963	4,653	8,178	10,237	2,38	1,46	9,21	1,46	0,095	286,97
Gestational syphilis**	Brazil	0,550	0,690	0,872	1,082	1,114	1,144	0,506	0,86	0,24	0,82	0,45	0,928	-4,65
	Paraná	5,747	7,332	8,164	9,302	9,419	9,666	3,807	7,99	1,52	6,74	4,14	0,743	-15,64
	Ponta Grossa	11,342	17,560	20,294	14,814	18,420	28,621	37,262	16,49	3,49	32,94	6,11	0,130	99,76

Rate = *Standardized incidence rate (100 thousand inhabitants) for confirmed cases with population of the state of Paraná as the standard/**Standardized incidence rate (1000 live births) for confirmed cases with live births in the state of Paraná as the standard population.
